# The Next Generation of Microbial Ecology and Its Importance in Environmental Sustainability

**DOI:** 10.1007/s00248-023-02185-y

**Published:** 2023-02-24

**Authors:** Michael Lemke, Rob DeSalle

**Affiliations:** 1 Department of Biology, University of Illinois at Springfield, Springfield, IL USA; 2 Institute for Comparative Genomics, American Museum of Natural History, New York, NY USA

**Keywords:** Microbial communities, Environmental sustainability, Restoration ecology, Genomics, Culturing, Sample preservation

## Abstract

Collectively, we have been reviewers for microbial ecology, genetics and genomics studies that include environmental DNA (eDNA), microbiome studies, and whole bacterial genome biology for *Microbial Ecology* and other journals for about three decades. Here, we wish to point out trends and point to areas of study that readers, especially those moving into the next generation of microbial ecology research, might learn and consider. In this communication, we are *not* saying the work currently being accomplished in microbial ecology and restoration biology is inadequate. What we are saying is that a significant milestone in microbial ecology has been reached, and approaches that may have been overlooked or were unable to be completed before should be reconsidered in moving forward into a new more ecological era where restoration of the ecological trajectory of systems has become critical. It is our hope that this introduction, along with the papers that make up this special issue, will address the sense of immediacy and focus needed to move into the next generation of microbial ecology study.

## Introduction

Let us start with the basics. Ecology “is the study of the relationships between living organisms, including humans, and their physical environment; it seeks to understand the vital connections between plants and animals and the world around them” (ESA website: https://www.esa.org/about/what-does-ecology-have-to-do-with-me/). Restoration ecology is the “intentional activity that initiates or accelerates the recovery of an ecosystem with respect to its health, integrity and sustainability” [1]. One cannot understand or implement the latter without characterizing the former. Microbes include viruses and cellular microorganisms; thus, Bacteria, Archaea, and microscopic members of the Eukarya (e.g., Protista and Fungi) are a part of this massive biota that makes up the environment. It is arguable that multicelled Animalia and Plantae have, by definition, members or life stages that are microscopic in size and, thus, have some of the same characteristics as microorganisms (i.e., Placozoa [2] for animals and pollen for plants). In aggregate, these microscopic entities drive most processes in the natural world, including nutrient cycling, energy flow, as well as disease. In this paper, we attempt to comment mostly on microbial impacts on ecological restoration, but to do this efficiently, we need to dive into some ecology.

The formal roots of microbial ecology are traced back to the mid-nineteenth century with the work of Martinus Beijernick and Sergei Winogradsky, the latter noted as the father of microbial ecology [3]. As a subset of the larger field of ecology, the study of microbial ecology began as a study of the microorganisms in their environment and their interactions with each other. What has changed is the scale and scope of environmental data that are collected to address classic and novel ecological questions. The primary focus of microbial ecology in the past was on communities in soil and water, and those microbes living symbiotically around plant roots. The approach in traditional “macrobiotic” ecology was to collect data and design studies that relied heavily on random sampling and replication. We say “heavily” because while much of science recognizes the need to account for variability in nature, it can be confounding due to the complexity of variability. In addition, whereas experiments designed for the laboratory build in factors to control variability, organisms in the environment are exposed to many factors, varying on a gradient of conditions (as opposed to defined factors). The upshot, in this generalization, is that ecological studies often have large sampling components consisting of a good number of replications to help characterize “differences” between as well as within treatments.

## Next-Next-Generation Microbial Ecology

What we have observed in microbial ecology studies is that sampling is often reduced, not that the investigators were unaware of valid sampling approaches to ecological studies, but rather, there was a certain “excused” reduction that was accepted because the analysis of samples was complicated, expensive, and time consuming. Early in the exploration of microbes in nature, samples were taken and the measurable factors (e.g., temperature, organic matter content, pH) were quantified using replication. A problem that arose was the combining of samples into a composite sample from which an even smaller subsample was taken for species identification. This was done because (before massively parallel sequencing for species identity) DNA was extracted, the 16S region (or portion) was amplified, and the amplicons were cloned into competent *Escherichia coli*, making a surrogate of the natural community. Going for a few hundred clones was common and, as we know, largely not adequate to describe the complexity of microbial communities. This approach seemed acceptable at the time.

There is a carryover effect still evident in some studies, that is, a reduction of replication or the creation of a composite sample before performing high-throughput 16S amplicon sequencing. Today, extraction methods are rapid (more on this below) and the amount of information obtained from samples processed for DNA sequencing is substantially more copious than before. With high-throughput parallel DNA sequencing, another problem emerged—how do the data from a study consisting of output of thousands of operational taxanomic units (OTUs) become characterized by the investigator. This created a taxonomy problem that can be boiled down to what taxonomic level is appropriate for any given study.

The modern focus on the ecology of microorganisms takes into account nearly every habitat on earth and at a scale that ranges from the landscape level to the cellular level—if everything is connected, and, with reference to the biogeography of microbes in the work by Martinus Beijerinck and articulated by Lourens Baas Becking, “everything is everywhere, but, the environment selects” [4–7], then the job of sorting which microbes are active and functional from those found in a habitat remains a formable task. An interesting topic related to this concept is the question, “How do microorganisms find their way to everyplace?” and while significant work has been accomplished about bacterial movement in biofilm models (e.g. [8, 9],) and liquid (e.g., twitching, swarming) and we know that bacteria respond to signals (e.g., root nodule formation, quorum sensing, and photo-, chemo-, magneto- and other taxis), information is only now surfacing about the movement of microorganisms through more solid media, like soil and sediment pores (e.g., [3, 10]). Microbial movement is a topic deserving greater attention, not only to better understand microbial biogeography, but also to understand the essence of ecology—that everything is connected, and if so, microbes are an important part of that connection.

These topics reinforce the motivation for microbial ecologists to remain cognizant of the complexity of microbe interactions and responses that include more species and a plethora of abiotic factors that can affect microbes. This takes into account many factors as well as a broad time scale. Therefore, modern microbial ecology is more than a list of microbes in a community. It consists of their life histories and an impressive array of factors that include temperature, moisture, pH, salinity, oxygen content, and many more bits of metadata. Often, the perception of microbial communities is that they change quickly. Relative to most other communities, this is true; however, we should remain aware that not all members of the community will show rapid change in function or reproduction. In addition, population variation has been a part of many ecological studies—yet with microbes, variation is often overlooked, i.e., all bacteria individuals of species are the same. Yet, we know this is not true as different strains of species cause different responses in disease and even with the members of the culturable species from nature, not all individuals respond to culturing conditions [11].

To partially address the array of factors relevant or involved in the ecology of a community, multivariate analysis is often used to ferret out the predominate factors associated with population change. Essential to seeing adaptations to habitats are factors relating to growth, such as energy and nutrient assimilation transfers, which are directly relatable to the diversity and recalcitrance of carbon sources and availability of different nutrient forms. Community sampling will give indication of changes that may correlate with environmental factors but not necessarily have predictive value. To understand change, frequent sampling to capture the quick responders coupled with sampling on a habitat, or factor-specific, scale, will yield the most interpretable results.

### Microbes and Restoration Ecology

The first step in thinking about restoration of any ecological community is to characterize the members of that community. The ecology of every macroscopic and microscopic organism in nature is linked to members of the domains Bacteria and Archaea (formerly known as prokaryotes), viruses, and their function in the environment.

It has been stated that one does not deeply understand ecosystem function until one tries to rebuild or restore the ecosystem [12]. Thus, restoration ecology provides a test of our theoretical and applied understanding of the field of ecology—a field that has only scratched the surface of our understanding of the members of the microbial component and even less so of an understanding of diversity and function. But, there is another challenge associated with restoration ecology. Restoring ecosystems to an earlier, pristine state may be the goal in some cases, yet the goal is challenged, given the recent advancement of climate change. The development of thinking about restoring ecology to a former state has been better stated as restoration of the ecosystem trajectory [13–16]—yet still begs the question of where that trajectory is going in recent times. It is possible that we have entered an era of the development of functionally distinct, novel ecosystems, i.e., ecosystems by design. As this new era develops, it becomes even more important to describe the microbial world with the fast and slow populations in mind. We have a reason to believe we can be successful, for as the diversity of microbial communities already shows us, we have microbes that have survived billions of years in refugia throughout our planet. Because of the size and diversity of habitat, it is of the utmost importance that researchers sample appropriately to include undisturbed sites as well as sites that have received anthropomorphic modification.

We hope this special issue, then, represents a landmark from where other investigations can be launched. In this opening article, we hope to stir things up a bit by making some fairly unorthodox statements followed by justification for making them. In some cases, to some readers, these statements might seem obvious, but we feel it is important to make the obvious even more so. Here, we address several issues among them: (1) how to sample nature in restoration studies, (2) molecular signatures and the culturing of novel microbial taxa, (3) the utility of species concepts in the context of microbial ecology, (4) the ecology of microbes in time and space, and (5) building a microbial reference system for restoration. We first explore the reliability of methods used to generate ecological hypotheses about microbial communities.

### Reconsidering the High-Throughput Approach to Understanding Microbes in Nature

Genomics opened the door to discovering the diversity of microbes, which appeared to surprise a lot of people. However, technique development co-occurred with the initial discovery and understanding about how cells in nature were passed over or in other words, as scientists discovered more about microbes, they also found diverse cellular compositions or sizes that made them harder to detect by initial extraction methodology—fine for then at that point in time, but we suggest that the approach to the genomic approach to microbial ecosystem analysis should be reconsidered now.

There are five essential steps that are needed to recover a sample from nature and characterize the community: (1) field collection, (2) DNA isolation, (3) sequence generation, (4) informatics (organizing the data along taxonomic lines), and (5) ecological interpretation. Problems occur at all steps (see summary in Table 1), and it is the purpose of this section of the paper to describe these. Several of the papers in this special issue address the nuances of these major steps. In this paper, we augment these discussions with our own take on field collection and DNA isolation methods.

**Table 1:** Collection of environmental DNA for community analysis and considerations

	Step	Caveat
1	Collection issues: obtaining samples	Composite sampling overlooking microhabitats; storage conditions
2	DNA isolation	Cell lysis; contaminants
3	Sequence generation	
	a. Amplicon studies	PCR inhibition
	b. Shotgun studies	Difficult informatics
4	Bioinformatics	Different approaches will yield different results
5	Interpretation	Application of ecological background and knowledge

### Collection Issues

To better frame the results coming from a particular sample, one should sample as specifically as possible. Given the diversity of microorganisms and their vastly different adaptations for survival in nature, it is difficult to imagine any sampling scheme that captures representatives of all of organisms in a community or system and this presents cross-comparison problems.

The primary areas of concern in attempting to characterize a community through genomics are sampling (number of samples and scale of sampling), abundance of DNA templates in a sample [17], lysis of cells, condition and recovery of DNA [18], and contaminants (summary in [19]). Study design and sampling protocols are part of most ecosystem studies, and the design issues for microbial ecological studies have been considered by a number of publications [20–23].

These publications point to two aspects needing attention in microbial ecology studies: sample replication and scaling (i.e., microhabitat). In a perfect world, we would simply sample every individual in a community, but sampling bacteria on the cellular scale is not practical. Because the organisms being sampled are so small and the microhabitats that can define abundance and distribution are many, the current sampling is often of bulk soil or water that is far from specific. For instance, even within a soil aggregate or at the sediment–water interface where gradients in physical and chemical properties are quite prominent, conditions can go from aerobic (+ 200 mV) at the surface to anaerobic (− 400 mV) in a distance of about 1 mm (e.g., [24, 25], thus affecting other processes (e.g., [26, 27]). It is difficult not to sample overlapping microhabitats; however, discussion of the intent and shortcomings of sampling should be made clear. The current approach seen in many studies of collecting bacteria in a variety of habitats yields a great diversity of microbes. Best practices for sampling in ecological studies abound (e.g. [28],), and we will not review them here. Suffice it to say that before setting out to collect for a study, several considerations should be in mind when sampling microorganisms in nature to help achieve restoration goals.

First, sampling with replication is necessary. Initially, due to the cost of DNA extraction, cloning and sequencing made it excusable for limited samples to be processed. Often in microbial ecology studies, replicate samples were collected for physiochemical measurements, but then, samples were combined and/or a small subsample was taken for molecular biology work. Increased automation and refinement of techniques now exist, along with the computer programs and computing power to process great amounts of information, so increasing the number of sample replication and reconsidering the extent and number of the microbial habitat that defines a community will help to understand site variation. We encourage more sampling now that informatics can process the information. The last two sampling considerations (temporal and spatial issues) have to do with scale. If investigators are concerned about a microbial process that shows variation on a time scale of days or weeks, infrequent, seasonal sampling will not lend itself to insights for the ecology of the site. Sampling frequency needs to be appropriate for the rates of change that are the focus of the study. The other consideration is spatial—as with the time-scale sampling consideration, if one is investigating diversity, function, abundance, or other properties of a microbial association, consider sampling on the microhabitat level. For example, where it was once fine to take a scoop, core, “grab” soil sample, ecologists recognize microhabitats in soil (e.g., variation in oxygen in the top few millimeters of the lake sediment or within a soil aggregate) are lst in these kind of sampling methods. Thus, sample appropriately, if you wish to understand the interaction of microorganisms with ecology of the habitat already recognized by ecologists. Will this produce experimental design and sampling nightmares? Likely, yet the techniques in DNA extraction and the informatics available to process the information have emerged, thus making this a good time to begin to move beyond composite sampling.

How will replicate samples and sampling on the time and spatial scales of microorganisms in their habitat help advance restoration ecology? Think of it this way—in sampling water, rhizosphere, or soil grab samples, you are creating a picture of the microbial world superimposed on a macrobiotic backdrop. A fuzzy yet significant image has been produced. Increasing sampling on a microhabitat appropriate scale will increase the “pixel” density of the overall image, allowing for more accurate interpretation, and diminish misinterpretation. This is a work-intensive proposition, but what development in science is not?

### DNA Extraction Issues

The extraction of DNA from natural samples continues to be a complex process with trade-offs between rapid, standardized methodology and more thorough, time-consuming approaches. The challenge of collecting microorganisms “from nature” has taken scientists into nearly every habitat on earth, with many habitat-specific considerations. While this is admirable, have we really gotten “all of it” that is out there?

The diverse species that exist in nearly every habitat need a common approach to study that includes environmental DNA (eDNA) extraction from organisms with more recalcitrant cell walls and from those that adhere to other organisms or surfaces. Extraction methods that target cells suspended in solution may not lyse cells that adhere in aggregates and are embedded in a greater amount of extracellular polymer matrix. Increasing the severity of cell disruption, such as through sonication, is risky in that it may dislodge cells but also degrade exposed cellular components, including DNA. As extraction methods migrated from pure cell cultures to the search for microbes in nature, a simultaneous trend emerged to develop methods that would maximize laboratory bench efficiency across a wide range of samples. Commercial kits emerged that paradoxically served to standardize methodology with respect to steps in procedure and timing while moving identity of reagents and materials into a proprietary realm.

Several recent publications [29–39] have addressed the issue of DNA isolation in microbial community studies and suggest that much of the methodology that has been worked out for soil, sediment, and water can work well for DNA extraction from most environmental samples but with several caveats.

Water sampling presents the challenge of retrieving living organisms from liquid and therefore represents cell acquisition from a disperse collection matrix—essentially condensing a dispersed tissue onto a collection surface. There are some particle adhesion and PCR inhibitors (e.g., humic and fulvic acids) that will result in differential representation of microbes in a sample. One nearly unavoidable consideration is mechanical acquisition of cells through filtering. Consider what is being filtered and the implications from doing filtration. For instance, bacteria on aggregate particles have been shown to have high metabolic activity (e.g., [40, 41]) and have been thought to greatly contribute to a more “mobile” basis of the aquatic microbial loop [42]. The microbial loop, first described for marine systems [43], but later in freshwater and soils, describes a trophic pathway where dissolved organic carbon and nutrients can be transferred to the “macro” grazer food web through uptake and subsequent consuming by various microbes. From the start of the filtering process, the nominal pore size changes in that particles captured by the filter also “clog” the filter. While the cutoff is often 0.45 µm or 0.22 µm, the actual size is different. But on the other hand, picoplankton and nanoplankton are still often missed, as can be seen if 0.22 µm water is passed through 0.02-µm filters (i.e., filters used to capture viral particles). A sample spread plate culture will confirm that a culturable portion has passed through and given the dictum of the plate-count anomaly, one can be sure many other microbes have passed through the filter. Efficiently capturing all the microbial species from water samples should be a consideration in future studies. While some investigators already employ a strategy for use of multiple filters of various pore sizes, recommendation of more standard protocol may help to advance this field of study.

Underestimation of diversity can also come from the inability to separate some prokaryotic cells from inorganic particles. After they are separated, the ability to lyse those cells also becomes important. The approach to the problem can take into consideration chemical (i.e., enzymatic) and mechanical (i.e., vibration, particle collision) approaches. We discuss the following aspects of DNA isolation from soil and water community samples here. These aspects include issues relevant to inhibitors, enzyme pretreatment, and mechanical and abundance issues.

#### Kits

Using DNA isolation kits has been a huge advancement for processing eDNA and microbiome studies. They offer convenience and uniformity for one of the steps in a rather complex protocol. But do they fit the bill for the DNA isolation step? The following discussion might seem picayune to some, but unconsidered picky aspects of sample treatment could change conclusions about communities under study.

#### Inhibitors

In two of the most common natural media—soil and water—factors affecting DNA extraction and subsequent amplification include PCR inhibitors (e.g., humic acids) with molecular weights and properties like DNA having the capacity to shut down PCR by binding to Taq polymerase. In the early phases of environmental DNA study sampling, tried and true isolation methods (e.g., precipitation, competitive protein amendments) were adopted. As the field progressed, other steps were identified that could produce better results by dealing with PCR contaminants. This problem is lessened when using shotgun sequencing protocols but can be a confounding factor in any environmental microbial analysis of ecosystems.

#### Enzyme Pretreatment

Some kits and procedures have an enzyme treatment step, which often includes lysozyme (muramidase). Given proteoglycan is a common component of the cell wall, but to different degrees for Gram-positive and Gram-negative cells, hydrolysis of this layer can aid spheroplast development and/or cell lysis. However, the cell envelope, which has the cell wall as a component, can present many other challenges for acquiring DNA, which range from a slime-water buffer layer to non-proteoglycan compounds (e.g., mycolic acid; or many others). A “one size fits all” approach to prokaryotic DNA isolation is seldom done and, for that matter, on the community level, is very hard to accomplish. In general, the efforts to pretreat samples with enzymes have been reduced over time, given the diversity of the cell envelope. Improved extraction approaches can include higher levels of lysozyme and proteinase K, as well as the addition of achromopeptidase, chitinase, and cellulase [44–47]. As mentioned above, application of this pretreatment is appropriate for many laboratory cultures and will positively affect extracts from soil and water, but it will not solve the problem.

#### Mechanical Approaches

Separating a cell from a surface makes the extraction step more productive—the problem is microbes do a great job of sticking (e.g., charge attraction, mucopolysaccharides) and biofilm composition and communities can be very complex. Approaches to dislodging cells and lysing them at the same time included sample sonication, freeze–thaw membrane disruption, and bead beating of particles [48]. In early isolation protocols, physical manipulation of samples yielded some positive results, but the problem was that the greater the physical force to separate and/or lyse cells, the greater the likelihood that the DNA would fragment to a point of not being confidently usable. When pursuing development of this method, empirical measurement of cell lysis (i.e., DAPI counts) to length of time and extent of sonication energy can guide the process.

Nearly all methods of DNA extraction appear to work if there are abundant numbers of microbial taxa in the sample. In general, if there are adequate numbers of a species present, a DNA signature will be recovered, and recalcitrance of the cell wall is less of a factor with these approaches. It is safe to say that we have, in the last 20 molecular-intensive years of investigation, developed a picture (e.g. [49–54],) that favors the more abundant and misses the rare (i.e., the fewer the number of individuals from a particular species, the lower the chance of detecting with the more brute force method of cell treatment to liberate the DNA). When there are fewer cells, gathering DNA is more challenging [55], so combining physical, mechanical, and chemical lysis approaches to isolate community bacterial DNA can yield better results.

Here, the balance lies in whether there is enough mechanical disruption to break the cell wall without shearing the DNA, or creating spheroplasts through the dissolving of cell walls, which vary far beyond the Gram-positive and Gram-negative fundamental compositions. As mentioned by Luna et al. [17], future studies should not only integrate different DNA extraction procedures, but also explore the possibility of integrating two or more different genetic markers in order to increase our ability to detect the actual bacterial diversity in environmental samples. We also point out that while much has been learned from the non-cultured approach (which should continue), efforts to understand the culturable aspect of microbial ecology should be done as well.

How can revisiting DNA extraction issues advance restoration ecology? Simply by developing the full picture of members of the community. Removing DNA signature bias by inclusion of as many and as diverse microorganisms as possible gives insight to both community structure and function. This is important because microbial diversity is related to system diversity. Think of the rule of microbial infallibility, originally stated as “Somewhere or other some organism exists which can, under suitable conditions, oxidise any substance which is theoretically capable of being oxidized” [56]. Now, think about this from the organism level—that is, some substrates need to be acquired under recalcitrant conditions, and some organisms need recalcitrant conditions to use substrates for energy (i.e., oxidation) or dissimilative metabolism (i.e., e-acceptors). Arguably, these may not be exceptions or minor components of life on earth, much, for instance, are anaerobic, acidic, reducing, etc. Logically and empirically, we have found that organisms needing to live in harsh habitats have adapted, many with variations in the cell envelope, and these habitats can be large in some areas, or intercalated among less harsh habitats (ex., soil depth or a soil aggregate mentioned above). (Note: what people often overlook is that what is considered harsh by the human is not harsh for the organism that adapted to grow in that microclimate, and even if not abundant, supports survival.) Without being more thorough in lysing cells, we are missing part of the picture even if that picture is composed of molecular signatures (i.e., culture independent).

### DNA Sequencing Issues

Whether sequence data are obtained by amplicon sequencing (e.g., using 16S genes as targets) or shotgun sequencing of the samples makes a big difference [57–60]. While interpretation of data sets done with the same sequencing approach might be cross-comparable, it is difficult to justify cross-comparison of shotgun results with amplicon results.

Not all amplicon-based studies generate representative assemblages of 16S rDNA [61–64] or other “barcoding” genes as it appears that some primers do a better job of amplifying broader arrays of taxa than others. For instance, Thijs et al. [64] point out that the primer pair 341f/785r appears to be optimal for soil- and plant-associated bacterial microbiome studies over several others. Multiplexing [65, 66] and hybridization that capture enrichment approaches [67] may solve some of these problems. In the end though, shotgun sequencing and the more difficult bioinformatic data analysis that accompanies shotgun sequencing could be a general solution to evening out the representation of organisms in environmental samples caused by DNA sequencing anomalies. The implications for restoration ecology are about the same as those for DNA extraction discussed above—that is, lack of consideration of DNA sequencing biases (e.g. [68],) will impact the diversity measures of a community blurring a detailed/clearer picture of the community.

### Using Molecular Signatures to Culture Species

We need to return to culturing microbial strains and species to understand the physiological, biochemical, and ecological nature of communities more fully.

The importance of biology in the study of microbial ecology cannot be ignored—that is, how and where do microorganisms live in nature? It is difficult to disagree, even 20 years since the writing, of the statement “We are grossly ignorant of bacterial life on earth” [69]—but we are making progress. Here, we promote areas that will help lessen ignorance. While culturing of microorganisms provided insights to the physiology and pathology of bacteria and protists, only a small percent (estimates range from about 1 to 2% [69, 70]) of the microbes found in nature could be grown in the lab, a calculation nearly any laboratory studying soil and water can make and was formally documented as the “great plate count anomaly” in 1985 [70]. The validity and reasoning behind the calculation and implications are much discussed in the literature (e.g. [71]).

The most rapid advances in describing microorganisms taken from nature began in the mid-1980s pioneered by Norman Pace, known by some as the Father of Microbial Ecology [72] and perhaps better distinguished as the Father of Molecular Microbial Ecology. What Pace effectively accomplished was to sidestep a 125-year-old, fundamental yet incredibly important approach to the study of microorganisms—culturing. Pace’s approach was to extract DNA and selectively sequence a gene linked to mapping out the phylogeny of Bacteria and Archaea. While yielding a great and useful amount of information, this approach set up two problems for the study of ecology: (1) Organisms retrieved from samples were destroyed upon analysis, and (2) it produced a reliance on the definition of species that was solely molecular.

While culture-free studies have produced a sea of change in how microbial ecology is done, we suggest the need to return to culturing of microorganisms obtained from the field, as it can rightly be said “to grow them is to know them.” The admission that a few species were culturable may have served as an excuse to use molecular techniques as opposed to a challenge to creatively try new approaches to culturing species. It may be argued that the field of study could not come back to a culturing approach without first going through the non-culturing approach. Comprehension of the extent and depth of 5 × 10^30^ cells of microbial life on earth was simply not known and, even when better described now, remains almost unfathomable. Be that as it may, culturing is being looked at more favorably (e.g. [73–81], and is an approach that should continue.

The contribution to ecology, and science, to grow more and different microorganisms includes the classical requirement of having a pure, archived culture (i.e., biological archetype) of the species in a collection. Also, by being able to grow microorganisms, insights are gained into the physiology (i.e., function) and habitat preferred by the microorganism. As for the impact on restoration ecology, better culturing techniques will result in a more precise and complete picture of biodiversity not only taxonomically, but also in function. Why? Having a culture allows for examination of the real biology and physiology of the organism. In other words, culturing advances two important aspects of restoration: (1) it is essential for targeting organisms included in reconstruction/restoration of damaged ecosystems, and (2) it allows us to observe growth, physiology, and reproductive behavior of more members of a community and an assessment of the presence of growth of organisms under target conditions.

### Post-sequencing Informatics and Interpretation

The goal of microbial ecology studies at first was to characterize diversity. Now that researchers have accomplished a good deal of this initial objective, many more recent studies have turned to functional and higher-level problems such as ecosystem function at the molecular level and ecological community restoration the subject of this special issue.

Currently, our analytical tools and theory that are in place for processing the large amount of sequence data in microbial ecology studies are thorough yet many studies use diversity measures to fuel analyses, base assumptions (i.e., high diversity = stability), and benchmark comparisons. While these tools have been useful in making some inferences about ecosystem content and, in some cases, how ecosystems work, a set of tools focused on more functional aspects of an ecosystem needs to be put in our microbial ecology and restoration toolbox. What might these tools be?

Consider the following example: if we have a process in a microbial community or an ecological niche, say, sulfate reduction, it often occurs in a very narrow range of conditions (i.e., highly reducing; sulfate occurrence, anoxic); if the reader does not like sulfur, then pick nitrogen or some other microbial process. Given that nutrient cycling has ecological value (i.e., provides ecosystem services), one should expect to find less diversity for this process—not more, in specific microhabitats. At first blush, this seems obvious (and it should be); one finds a specific enzyme for most specific processes, but to understand the biochemistry of the process, one cannot just put the whole community in a test tube (or even a whole organism), hydrolyze it, sequence it, and expect to understand each mechanism. Rather, to understand the phenomenon, the community (or individual) needs to be teased apart reaction by reaction.

What other analytical ecological methodologies exist that are potential ways to get at ecosystems? Some researchers have argued that multitrophic approaches in community ecology are essential to achieve full ecosystem understanding [82]. If so, then the trophic dynamics of viruses as food need attention. In the context of how diversity is involved in multitrophic approaches, Karimi et al. [83] argue that diversity indices lack sensitivity; therefore, subtle damage to systems (low level but consistent pollution) goes undetected. They conclude that looking at networks (i.e., connections) is the way to go. However, this approach may be confusing as multitudes of connections are hard to track and there is the problem of “strong” connections vs “weak” connections. Shade [19] may have said it best “I argue that diversity without context provides limited insights into the mechanisms underpinning community patterns.”

What does this mean in the context of microbial ecology restoration? As we said earlier in this communication, if we sample on a broad scale, we get complex and often confusing evidence. Yes, there is high species richness quite likely because the sampling strategy cut through various oxygen contents, pH levels, and other physical gradients. The organisms in the collected samples depend on a host of different substrates for energy and other processes for terminal electron acceptors (TEA), etc. As Shade [19] implies, if managing and restoration efforts have diversity as an endpoint, we could do it. For instance, by adding many different carbon forms, we could measure higher diversity and the endpoint would be reached. But have we done anything to better manage the system for recovery or even better stability? More thought needs to be put into how diversity and restoration intertwine.

### Species-Level Considerations

A generalized microbial species concept may not be worthwhile in community ecology studies as species can and will become somewhat less relevant when considering microbial communities. This phenomenon is even more acute when applying the results of genomic approaches to microbial ecology like restoration biology. Ecological or physiological functions derived from microbial genome sequencing might be a better guide to how to view diversity in microbial ecology.

Describing the microbial community using DNA has offered fantastic benefits in a world where the organisms that had been described were relatively few, since attempts to initially characterize communities was by culturing. Focusing on inferences made from 16S ribosomal DNA provided a huge jump in discovery, given the molecular dissimilarity among organisms was correlated to an evolutionary divergence that yielded many distinctions not seen by non-molecular methods. Species-level relationships were the goal of such studies. Bacterial species definitions though are slippery [84]. However, there has been some positivity about delimiting and describing bacterial species [85, 86] and see below. On the positive side, naming the massive number of bacterial and archaeal species needed to work at this level may not be insurmountable [87]. Another positive is that the deposition of information at this level is in a universal, searchable database (i.e., NCBI [88]). However, acquisition and archiving of DNA sequences has brought us to no less than two difficulties as the field progresses.

First, the large number of nucleic acid fragments sequenced and linked to brief descriptions in the database helps with the classification of organisms but, in many cases, not to the level of species. While the approach can work for categories at the generic and family levels and higher, there is a breakdown on the species level, especially in organisms that are putatively closely related. While there is some evidence that bacterial species boundaries are real using average nucleotide identity (ANI) [89, 90], the difficulties with distance-based methods to delimit species in general and bacteria in particular need to be reconsidered [91].

Second is that while the definition of prokaryotic species can be supported by DNA sequencing [89, 90], we suggest that the actual meaning of a bacterial or archaeal species will stem from its function in the environment whether that environment is soil, water, human, or other. In bacterial species, the functional niche [92, 93] can also define the species classification and should be based on the evolution of bacteria and archaea to acquire energy. Leading to our suggestion above that microbial function, physiology and biochemistry may be more appropriate guides for classification at the community level. In other words, we may not need species level descriptions to proceed with microbial environmental ecology. The problem is that if we progress with a more physiological-biochemical framework, we decouple the traditional way of looking at communities (species diversity–based approaches) from these non-species-based approaches. Linking the two in the future will also be important.

The implications to restoration ecology lie in the importance of this topic to ecology as a field of study and, even, to science in general. Defining the functional niche is difficult but necessary, but here, either we are tasked to taking what we know from cultured organisms and developing the multiparameter matrices that define function (under different circumstances) or we need to do in-depth analysis of the organism in nature. Studying the natural history of microbes has begun (e.g., [52, 94], and there is a lot left to do. A major complaint by many microbial ecologists is that adding this complexity of analysis seems an insurmountable task—to understand the function of hundreds of thousands of microbes in a community is a problem in complexity that we simply do not have the tools to do. But, we do have available background that could serve as the basis for developing a more complete theory to approach the more important questions in microbial community ecology and restoration. One needs to look no further than Bergey’s manual for a sound background for understanding function; true, the manual is based on culturable organisms, but it is filled with a table after a table of characteristics and enzyme reactions (i.e., function)—interpretable as a large matrix of function and thus a start.

### Building Reference Systems for Restoration

Saving samples and communities in a collection, preferably a cryo-collection, is certainly a worthwhile endeavor for the future study of microbial ecology.

Scientists value specimen-based collections to advance the study of biology and have made species-level collections of culturable bacteria. In fact, having a culture of an entity is necessary for its proper description as a new species. Microbial culture collections preserve species identity and offer viable specimens for subsequent study. We have known for some time that a “culture plate anomaly” exists; few soil bacteria and aquatic bacteria have been cultured and identified by culture-independent techniques [11, 83, 95–100]. Application of standard methods for long-term storage and archiving of microbes on the community or assemblage-level, however, have seldom been practiced and rarely researched [101–103]. This is not surprising because bacterial cryopreservation has been dependent upon culturing methodology. Since  about 1% of the number of bacteria from nature [95, 104] is culturable, cryopreservation of bacterial assemblages should become an important endeavor. Because we feel that this archival step is of utmost importance for the future of microbial restoration ecology, we look in detail at some of the nuances of the endeavor.

**Fig. 1:**
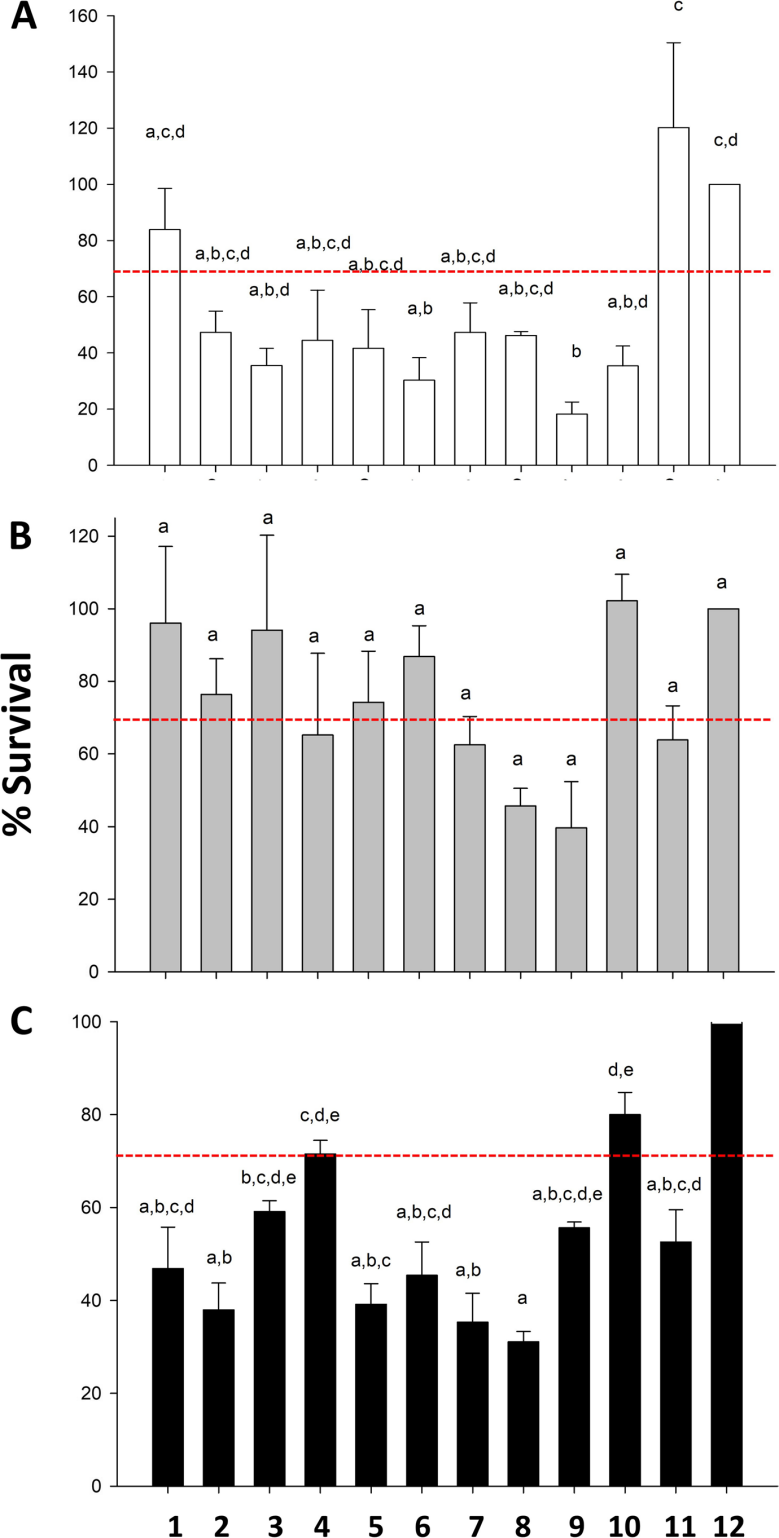
Comparison of cryopreservation methods for microbial assemblages. The percent survival of Bacteria (formerly Eubacteria) (**A**), Gammaproteobacteria (**B**), and Betaproteobacteria (**C**) after cryopreservation. Percent survival is plotted for 10 different cryopreservation methods. The cryopreservation of microbial assemblages was allowed to incubate at − 180 °C for 6 months before slowly thawing and then assessed by culture-dependent and culture-independent techniques. The treatments are as follows: 1, freeze with trehalose; 2, freeze with DMSO; 3, freeze without cryoprotectant (CPA); 4, Mr. Frosty (MF) with trehalose; 5, MF with DMSO; 6, MF without CPA; 7, lyophilize with trehalose; 8, lyophilize with DMSO; 9, lyophilize without CPA; 10, trehalose only immediate analysis; 11, DMSO only with immediate analysis; 12, no method, no CPA with immediate analysis. Treatments 10, 11, and 12 served as controls. Red line indicates 70% survival; the results of the cryopreservation experiments indicate that different phyla react to cryopreservation in varied fashion. (see text) (data from and are available at [110]; used with permission)

It is not known how many members of the microbial assemblage from an environmental sample survive preservation and therefore elude possibilities for future study and culturing [83]. Because of various osmolarity needs, cell size differences, and variation in cell wall composition in bacterial assemblages, it is likely no method of cryopreservation will be adequate for safe storage of all cells (discussion in [105]), and indeed, Fig. 1 shows this for three taxa. Commonly used storage techniques (e.g., desiccation, immersion in mineral oil, and lyophilization) have been proven to be short-term solutions [106, 107]. It is well known that samples stored at − 20 °C are subject to protein and lipid changes as well as cell fracturing [106]. Storage at − 80 °C is common (e.g. [108],) and may be appropriate, but rarely has been tested for long-term effects. The use of liquid nitrogen is advantageous because it sustains temperatures below − 150 °C, is not subject to power outages as are electric freezers, and helps reduce cellular physiochemical changes. Controlled freezing of samples, such as the Mr. Frosty technique (see Fig. 1) that decreases the storage temperature at a rate of about 1 °C/min, allows for cryoprotectants to permeate the cell, which allows cellular changes that help increase survival upon thawing [109].

The addition of cryoprotective additives (CPAs) often increases the recoverability of organisms by decreasing the freeze-fracturing effects of water [111]. Two common CPAs, dimethylsulfoxide (DMSO) and glycerol (G), have been effectively used to preserve eukaryotic and some microbial cells. DMSO rapidly penetrates the cell wall and cell membrane of cells within 30 min with concentrations varying widely from 1 to 32%, depending on the organisms being preserved [111]. Glycerol, typically around 10% concentration, is a common CPA used in microbiology and also rapidly penetrates the cell wall and cell membrane [111, 112]. In Fig. 1, we present results from work done by the authors and members of their labs to compare cryopreservation methods of cultured and uncultured samples. It appears that lyophilization is an inferior method of preservation compared to flash freezing and controlled freezing (Fig. 1). Figure 1 also implies that multiple preservation methods may be needed to adequately implement cryopreservation of communities for storage, but certainly more work needs to be done.

## On Restoration Ecology and the Human Microbiome

We began this essay by explaining that we hoped to lend perspective and constructive advice about the next generation of discovery in molecular microbial ecology and its potential to help restore degraded natural ecosystems. As we end our exploration of this topic, let us finish with a stupendous development that did not exist when molecular microbial ecology began—its expansion—and that is the description and application of knowledge that has come about by examining the human microbiome. Using the human microbiome as an analogy, we can cut through much confusion as to what is possible now and what has yet to be developed for microbial restoration ecology. As one thinks about applications in restoration ecology, we can substitute what we know for the human microbiome as an example for an ecosystem. We could of course complicate the comparison by considering the microbiomes of the many organisms that have been studied to date, but let us keep this analogy simple for now.

One of the first things to come out of the human microbiome exploration were some generalities. One item that surprised many non-microbiologists was that there were 10 × more microbes in and on a human than there were cells (eukaryotic) of the human body – a “fact” since contested but still a generality that is true in general even if exact order of magnitude is under debate. Let us start here by pointing out the analogies. At the level of ecosystem, there are some generalities as well. For instance, in 1 ml of nearly any source of freshwater, there are roughly 1 million bacteria (take or give an order of magnitude). This is surprisingly consistent. Likewise, the percent culturable is nearly always the same [11]. We can appreciate that the low proportion of culturable bacteria may be due to our own lack of originality and tenacity to tackle the question on a large scale, but why are there always roughly a million bacteria/ml of freshwater? Have bacteria reached a carrying capacity? Are these organisms sloughed off from decadent biofilm? One would expect, given the heterogeneity of an ecosystem, to see more variation not only in number, but of those cultured.

It is coming to light that the human microbiome is important to health and that the metabolism and relative abundance of the microbial community is tied to the health of its host. A variety of supplements, or probiotics, is being offered to contribute to the microbial ecosystem health of the human. If for no other reason, here is an example of why culturing (i.e., determining the physiology of a species) and population interaction should be studied more intensively in human as well as restored ecosystems. The concept of community inoculum has transcended both environments (e.g., fecal implants; root/rhizosphere soil-root plugs as opposed to plant seeds only).

The current approaches in human health and ecosystem health have no need to be lost to usher in insights from the microbial world. Restoration ecology calls on an understanding of the ecosystem in order to restore it. Here, application of genomics in microbial ecology can favor outcomes and reach goals, even in a changing climate.

## Conclusions

One of our major recommendations that may strike some as difficult to implement is that researchers should consider archiving samples taken for ecological analysis. Currently, most biological collections are based on single organisms (as an example, one need to look no further than the contents of a museum drawer in an entomology department). Some medical collections focusing on a specific tissue or blood of individuals are linked primarily to the individual, but such collections also include communities. Many researchers are now taking advantage of such collections to explore mostly for viral diversity, but the initial purpose of storing the tissue or blood in the first place was for future reference to the collected individual. We are calling for more directed sampling, storing, and archiving of microbial communities in museum collections that will be of invaluable future use in understanding the biology of restoration.

Our second unorthodox (but not entirely original; see [113]) suggestion is that we need to break away from the “tyranny” of microbial species designation and diversity measures. We are not suggesting that diversity measures up to now have not been useful—they have. Sequencing of DNA for species counts is a start. Whole genome sequencing, even if outside of the objective and budget of current studies, may be a possibility in the future. We are suggesting, however, that diversity is usually not the single goal of restoration, and so, other methods of characterizing an ecosystem and its restoration are needed.

Moreover, we suggest that we need to return to culturable techniques to really make greater progress in these endeavors. We point out that human microbiome studies have gone down the same road. Because human microbiome studies are done for more medical and therapeutic reasons, a different set of tools for that kind of research has been developed that should be transferable to ecosystem community ecology. Conclusions about probiotics and microbial transfer therapies are interesting examples of real-world applications of using knowledge from the human microbiome. In the analogy, we developed in this paper that a community slated for restoration is like a microbiome and the restoration measures are like probiotics.

We can think of no better way to conclude this essay than with a quote from Stubbendieck et al. [114] who summarize a novel integrated approach to microbial ecology in the following passage: “By breaking down and investigating communities over a wide range of scales, we are better able to understand fundamental principles of bacterial ecology. This includes deciphering the mechanisms of pairwise interactions, as well as identifying the species composition of a bacterial community. Through the integration of complementary experimental and theoretical approaches, the underlying foundation of dynamics in larger scale communities is revealed. Moving forward, the application of both approaches will be instrumental in garnering new insights into how bacterial communities influence all facets of lives on Earth.”
